# *Pseudomonas aeruginosa* Microcolonies in Coronary Thrombi from Patients with ST-Segment Elevation Myocardial Infarction

**DOI:** 10.1371/journal.pone.0168771

**Published:** 2016-12-28

**Authors:** Gorm Mørk Hansen, Daniel Belstrøm, Martin Nilsson, Steffen Helqvist, Claus Henrik Nielsen, Palle Holmstrup, Tim Tolker-Nielsen, Michael Givskov, Peter Riis Hansen

**Affiliations:** 1 Department of Cardiology, Herlev and Gentofte University Hospital, Copenhagen, Denmark; 2 Costerton Biofilm Center, Department of Immunology and Microbiology, Faculty of Health and Medical Sciences, Copenhagen University, Copenhagen, Denmark; 3 Section of Periodontology, Department of Odontology, Faculty of Health and Medical Sciences, Copenhagen University, Copenhagen, Denmark; 4 Department of Cardiology, Rigshospitalet University Hospital, Copenhagen, Denmark; 5 Institute for Inflammation Research, Department of Infectious Diseases and Rheumatology, Rigshospitalet University Hospital, Copenhagen, Denmark; 6 Singapore Center on Environmental Life Sciences Engineering (SCELSE), Nanyang Technological University, Singapore; Universiteit Gent, BELGIUM

## Abstract

Chronic infection is associated with an increased risk of atherothrombotic disease and direct bacterial infection of arteries has been suggested to contribute to the development of unstable atherosclerotic plaques. In this study, we examined coronary thrombi obtained *in vivo* from patients with ST-segment elevation myocardial infarction (STEMI) for the presence of bacterial DNA and bacteria. Aspirated coronary thrombi from 22 patients with STEMI were collected during primary percutaneous coronary intervention and arterial blood control samples were drawn from radial or femoral artery sheaths. Analyses were performed using 16S polymerase chain reaction and with next-generation sequencing to determine bacterial taxonomic classification. In selected thrombi with the highest relative abundance of *Pseudomonas aeruginosa* DNA, peptide nucleic acid fluorescence *in situ* hybridization (PNA-FISH) with universal and species specific probes was performed to visualize bacteria within thrombi. From the taxonomic analysis we identified a total of 55 different bacterial species. DNA from *Pseudomonas aeruginosa* represented the only species that was significantly associated with either thrombi or blood and was >30 times more abundant in thrombi than in arterial blood (p<0.0001). Whole and intact bacteria present as biofilm microcolonies were detected in selected thrombi using universal and *P*. *aeruginosa*-specific PNA-FISH probes. *P*. *aeruginosa* and vascular biofilm infection in culprit lesions may play a role in STEMI, but causal relationships remain to be determined.

## Introduction

Atherosclerosis (AS) is a chronic inflammatory disease, and vulnerable atherosclerotic plaques can rupture or erode, which can result in luminal thrombus formation and critical end-organ ischemia, e.g., stroke or myocardial infarction [1,2]. While a number of traditional risk factors for AS, such as hypertension, dyslipidemia, diabetes mellitus, obesity, and smoking are well-established, it is also widely accepted that other chronic inflammatory diseases (e.g., rheumatoid arthritis, psoriasis and chronic inflammatory bowel disease) and infectious diseases are also independently associated with manifestations of AS [3–8]. Direct infection of the arterial wall has also been proposed as a contributor to inflammatory plaque destabilization and thrombotic complications [6, 9]. In particular, there is an increasing interest in the potential role of periodontal pathogens in AS, e.g., *Porphyromonas gingivalis*, *Prevotella intermedia* and *Aggregatibacter actinomycetemcomitans* [10–12]. However, many other infectious agents may play a role in AS including *Chlamydophila pneumoniae*, *Helicobacter pylori*, cytomegalovirus, influenza A virus, and *Pseudomonas* species [6, 13, 14].

While there are several reports on isolation of bacterial DNA from atherosclerotic tissue only a few recent studies have detected bacterial biofilms within atherosclerotic lesions [6, 13, 15]. In biofilm infections, microcolonies of bacteria become encased in a protective extracellular matrix consisting mainly of polysaccharides, proteins and DNA [16, 17]. Biofilms are notoriously resistant to antibiotic treatment and host immune defenses, and can result in chronic infections including, for example, wound infections, middle ear infections, foreign body-associated infections, and periodontitis [16]. Coronary thrombi aspirated from patients with ST-segment elevation myocardial infarction (STEMI) during primary percutaneous coronary intervention (PCI) have also been demonstrated to contain bacterial DNA [18, 19]. In addition, the aspirated material contains fragments from the lipid rich plaques of culprit coronary artery segments and PCI with thrombectomy can hence serve as a method of *in vivo* sampling of coronary thrombus and plaque material [20]. STEMI which is typically manifested by acute chest pain and characterized by STE in the patient’s electrocardiogram, is usually caused by acute thrombotic occlusion of a major coronary artery and (if untreated) is associated with considerable mortality and worsened long term prognosis.

In this study, we hypothesized that bacterial infection of coronary plaques, e.g. with oral pathogens could be demonstrated by performing a microbiome study of coronary thrombi aspirated from patients with STEMI, and that presence of intact bacteria, potentially in the form of biofilms could be confirmed by microscopic examination. Since bacterial translocation is likely to occur via circulating blood, we also collected arterial blood samples as controls. We analyzed aspirated thrombi and arterial blood from patients with STEMI using next-generation sequencing to obtain taxonomic information on recorded DNA reads. In addition, we examined thrombi with the highest relative abundance of *Pseudomonas aeruginosa* DNA with peptide nucleic acid fluorescence *in situ* hybridization (PNA-FISH) with universal and *P*. *aeruginosa*-specific 16S rRNA probes to detect intact bacteria and bacterial biofilms.

## Materials and Methods

### Participants

We enrolled 22 patients with STEMI referred to one tertiary cardiology center (Copenhagen University Hospital, Rigshospitalet, Denmark) for primary PCI, and where thrombectomy was performed at the discretion of the invasive cardiologist. Patients were excluded if they were unable to give informed consent, e.g., due to cardiac arrest or cardiogenic shock, or if they presented with clinical and biochemical signs of active infection, e.g., fever, productive cough, dysuria or visibly infected ulcers with markedly elevated C-reactive protein (CRP) levels and leucocyte counts. Also, patients were excluded if they had received systemic antibiotics, or/and immunocompromising treatment, e.g., chemotherapy or immunosuppressive treatment within a period of three months prior to admission. Baseline characteristics were recorded and periodontal status was assessed by interview and a standardized questionnaire which generally has acceptable validity in determining periodontal health [21]. The interview included questions regarding number of remaining teeth, frequency of gum bleeding during oral hygiene, and periodontal disease diagnosed by a dentist. Self-reported periodontal disease was only registered, if the patient reported that their dentist at the latest visit prior to the STEMI had informed him/her of the presence of periodontitis.

The study was approved by The Regional Committee on Health Research Ethics of The Capital Region of Denmark (protocol number H-2-2012-154), and written informed consent was given by all participants.

### Sample collection

The thrombi were sampled during primary PCI using standard thrombectomy catheters (Export^®^ AP Aspiration Catheter, Medtronic, Minneapolis, MN, USA). Each thrombus was aspirated into a sterile syringe, and the content was emptied into the collecting basket supplied by the manufacturer. Here, the material was rinsed with heparin and retrieval of solid fragments was visually confirmed. The collecting basket was then immediately placed in a sterile container, sealed, labeled, and frozen at -80°C until analysis. An arterial blood sample was drawn from the femoral (n = 17) or radial (n = 5) artery sheath, transferred into a heparinized collection tube and stored with the thrombus sample.

### 16S polymerase chain reaction and next-generation sequencing

The samples were thawed on ice, and all handling was done in laminar flow bench using sterile equipment. Once thawed, each thrombus was cut in two sections: One section was placed in a 10% formalin solution for fixation before paraffin casting. The second piece was mechanically homogenized in 50 μL molecular grade water (Sigma-Aldrich, St. Louis, MO, USA), and transferred directly as template into a pre-prepared polymerase chain reaction (PCR) tube containing ready master mix. Similarly, the thawed arterial blood from the same patient was pipetted into 50 μL molecular grade water, vortexed and transferred to a different PCR tube with identical PCR master mix. Using high precision weighing (Sartorius ED scale, Sartorius, Göttingen, Germany) the weight of each thrombus before homogenization was registered. Comparable amounts of blood and thrombus could then be analyzed by pipetting 1 μL blood per 1 μg of thrombus. A nested PCR protocol was followed using primers annealing to the bacterial 16S rRNA gene. Master mix for both the first and second reaction consisted of Phusion Blood Direct Master Mix (Thermo Fisher Scientific, Waltham, MA, USA) which contained buffer solution with dNTP mix, proofreading Blood Direct Polymerase II enzyme stable to inhibitors in blood, and 0.25 μM reaction specific primers. For the first reaction, the primers used were 337F (5’- TCC TAC GGG AGG CAG C-3’) and 1391R (5’- GAC GGG CGG TGT GTR CA-3’). The thermo-cycle protocol included 5 min denaturing at 98°C followed by 30 cycles of 5 sec denaturation at 98°C, 5 sec annealing at 63°C, and 15 sec extension at 72°C. Final extension was at 72°C for 1 min. Debris was pelleted using tabletop centrifugation and the supernatant from the first PCR product was used in a 1:100 dilution as template for the second reaction. For the second reaction, the PCR primer pair was 515F (5’-GTG CCA GCM GCC GCG GTA A-3’) and 806R (5’-GGA CTA CHV GGG TWT CTA AT‘-3). The same thermo-cycle protocol was used for the second reaction but with annealing temperature 58°C for 15 cycles. Mock PCRs were used as true negative controls.

Sequencing of PCR products was performed by Beijing Genomics Institute that was blinded to the samples and provided DNA purification, amplicon library preparation (16S v4) and sequencing performed on Illumina PE300 MiSeq System (Illumina, San Diego, CA, USA) according to standardized guidelines [22].

### PNA-FISH

The five thrombi (male n = 3, female n = 2) with the highest relative abundance of *P*. *aeruginosa* DNA were analyzed with PNA-FISH. Each formalin-preserved thrombus piece was cast in paraffin. From the paraffin cast, six 2 μm thick serial sections were cut and placed on two microscope slides with three sections on each slide. One slide was incubated with a *P*. *aeruginosa* specific 16S PNA-FISH probe and the other with a universal 16S PNA-FISH probe. The samples were deparaffinated according to standard protocol (Xylene 2 x 5 min, 99.9% EtOH 2 x 3 min, 96.5% EtOH 2 x 3 min, MilliQ 3 x 3 min) and incubated with PNA-FISH probe for 90 min at 56°C [23]. The probes used were a fluorescent TexasRed (TXR) conjugated universal bacterial 16S rRNA probe (BacUni CP0054TxR, AdvanDx, Woburn, MA, USA) and a fluorescent TXR conjugated *P*. *aeruginosa*-specific 16S rRNA probe (*P*. *aeruginosa* ref. 102307TxR, AdvanDx, Woburn, MA, USA). After incubation with PNA-FISH probes, the samples were counterstained with a 3 μM DAPI-solution (4',6-diamidino-2-phenylindole [DAPI], Thermo Fisher Scientific, Waltham, MA, USA) for 15 min at room temperature and finally rinsed with PBS, air-dried and fixated with antifade mountant (ProLong^®^Gold antifade reagent, Life technologies, Eugene, OR, USA) [23]. The slides were examined using confocal laser scanning microscopy (LSM780, Zeiss Microscopy, Oberkochen, Germany). Objective used for imaging was 63x/1.4 Oil. Software for processing was Zen Lite version 2.3 (Zeiss Microscopy, Oberkochen, Germany).

### Data analysis

Sequences were clustered to operational taxonomic units (OTUs) at 97% sequence similarity and taxonomic ranks were assigned to OTU representative sequences using the Ribosomal Database Project (RDP) [24]. Subsequently, the sequences were blasted against two different 16S reference databases (Greengenes and Human Oral Microbiome Database [HOMD]) for genus and species specific identification [25, 26]. Alpha diversity was compared between the two sample groups (thrombi versus arterial blood) using Chao-index, Shannon-index, and Simpsons-index. Comparison of relative abundance at genus- and species level was performed using Mann-Whitney U test with Benjamini Hochberg correction for multiple comparisons. Software R version 3.0.3 was used as statistical software, and Graphpad Prism version 7and MultiExperiment Viewer version 4.9 were used as graphic software.

## Results

### Patient characteristics

The baseline characteristics are shown in Table 1. The study included 22 patients with STEMI who underwent thrombectomy during primary PCI. Mean age was 61±11.5 years and 73% of patients were male (Table 1). Smoking and hypertension were the most prevalent cardiovascular risk factors (both reported in 59% of patients), and 47% of patients reported known periodontal disease (Table 1). All patients were discharged after an uneventful in-hospital course.

**Table 1:** Baseline characteristics of ST-segment elevation myocardial infarction study population.

	Patients with STEMI (n = 22)
Age, years, mean±SD	61±11.5
Sex, male/female, %	73/27
Hypertension, %	59
Hypercholesterolemia, %	32
Diabetes mellitus, %	5
Smoking, active or previous, %	59
Previous MI, %	14
Family history of IHD, %	41
CRP, mg/L, median (IQR)*	4 (1.2)
WBC, x10^9^/L, median (IQR)*	11 (3.6)
TnT max, ng/L, median (IQR)	4600 (6350)
LVEF† (n = 19), n	
>45%	14
30–45%	3
<30%	2
Coronary artery culprit vessel, %	
LAD	41
LCx	50
RCA	9
Periodontal status (n = 19)‡, %	
Self-reported PD	47
Edentulism	16
Regular gum bleeding	32

STEMI, ST-segment elevation myocardial infarction; SD, standard deviation; IHD, ischemic heart disease; CRP, C-reactive protein; IQR, interquartile range; WBC, white blood cell count; TnT, troponin T; LVEF, left ventricular ejection fraction; LAD, left anterior descending artery; LCx, left circumflex artery; RCA, right coronary artery; PD, periodontal disease.

*CRP and WBC at time of admittance.

^†^LVEF at time of discharge.

^‡^Periodontal status was assessed at 11±3 months post-discharge, at which time 1 patient had died and 2 were unavailable for follow-up.

### General findings from next-generation sequencing

For each of the 22 thrombi and 22 arterial blood samples, bacterial rDNA was amplified by the use of PCR. Four thrombus samples failed quality control prior to next-generation sequencing due to degradation or low DNA concentration (<5 ng/μL), and these thrombi and their corresponding blood samples were therefore excluded. Thus, DNA from a total of 36 samples (18 thrombi with corresponding arterial blood) was successfully sequenced, from which a mean of 23,316 (range 17,468–28,136) sequences were generated with an approximate length of 250bp. A mean of 88.5% (range 67.2%–97.1%) of these sequences passed subsequent library quality control and were eligible for further analysis [22].

### Comparable microbial diversity in thrombus and blood samples

Using 97% similarity as cutoff, a total number of 210 different OTUs were identified with a mean of 29 (range 10–89) OTUs per sample (Figure A in S1 File). No statistical difference was observed in the mean number of OTUs between thrombus (mean OTU = 28) and blood samples (mean OTU = 30).

Alpha diversity was tested by means of Chao-index, Shannon-index and Simpson’s diversity index, which all demonstrated similar diversities in both thrombi and blood (Figure B in S1 File).

### High relative abundance of DNA from *P*. *aeruginosa* in thrombus samples

**Fig 1:**
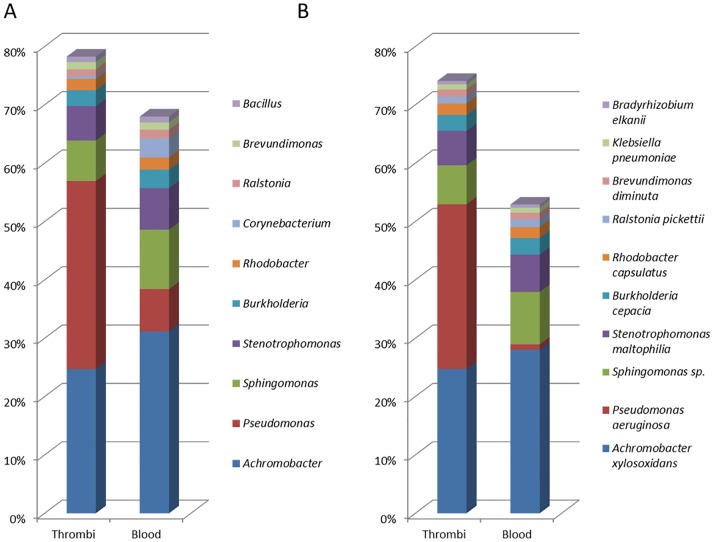
Predominant bacterial genera (A) and species (B) in aspirated coronary thrombi and arterial blood from patients with ST-segment elevation myocardial infarction. Data were based on Human Oral Microbiome Database (HOMD) annotation.

Based on the Greengenes annotation 90% of sequences could be identified at genus level with a total number of 96 bacterial genera identified (Table A in S1 File). The predominant genera identified were *Achromobacter* and *Pseudomonas*, corresponding to approximately 45% of total identifications at genus level (Figure C in S1 File). When blasted against the Greengenes database, only 10% of sequences could be identified at species level. Blast against the HOMD database resulted in identification at the genus level of 78% of sequences representing 54 different bacterial genera (Table B in S1 File). *Achromobacter*, *Pseudomonas*, *Sphingomonas*, *Stenotrophomonas* and *Burkholderia* were the predominant bacterial genera identified and accounted for 66% of the total bacterial identifications at genus level. The 10 most predominant genera and their relative abundance in thrombi and arterial blood samples are presented in Fig 1. Based on the HOMD annotation, 71% of generated sequences could be assigned at species level. A total number of 55 different bacterial species were identified with a mean of 14 (range 8–34) different species per sample (Table C in S1 File). The 10 most predominant species are shown in Fig 1. The dataset containing information on the numbers of DNA reads per identified species in each of the analyzed samples is available in the supporting information (S1 Dataset).

DNA from *P*. *aeruginosa* was detected in 18/18 thrombi with a mean of 5,563 (range 671 to 19,101) reads per thrombus which represented 28% of DNA from all species (mean 19,670 reads per thrombus) identified in the thrombi. In the arterial blood samples, DNA from *P*. *aeruginosa* was detected in 12/18 samples, but 7 of these 12 had only minute (≤ 5) number of reads. Overall, DNA from *P*. *aeruginosa* represented 0.9% of all species identified in the arterial blood samples (mean 179 [range 0–2,123] of 19,585 total reads). Accordingly, for *P*. *aeruginosa* both the relative abundance of DNA and the absolute number of DNA reads were >30 times higher in thrombi than in arterial blood (p<0.0001). In thrombi, DNA from *Achromobacter xylosoxidans* was detected in high amounts in both the thrombi and arterial blood samples and was not significantly associated with either thrombi or blood. In 10/18 patients, *A*. *xylosoxidans* constituted >20% of species-identified DNA in both thrombus and arterial blood from each patient. Remarkably, in 6/8 of the remaining patient samples, *A*. *xylosoxidans* constituted <0.001% of species-identified DNA in both thrombus and blood.

No significant correlation was found between self-reported periodontal disease, regular gum bleeding, or edentulism, respectively, and the relative abundance of *A*. *xylosoxidans*, *P*. *aeruginosa* or any other detected species.

### PNA-FISH results

**Fig 2:**
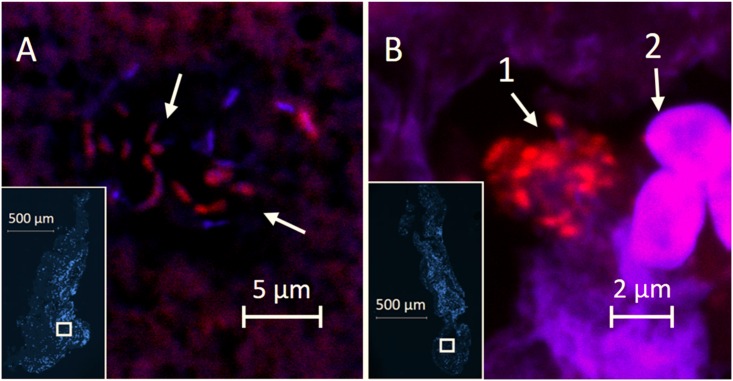
Confocal laser scanning microscopy images with PNA-FISH showing microcolonies of bacteria detected in coronary thrombi. Confocal laser scanning microscopy images with Peptide nucleic acid fluorescence *in situ* hybridization (PNA-FISH) of aspirated coronary thrombi from patients with STEMI. A: Magnified image of hybridization with a TexasRed (TXR) conjugated universal 16S rRNA probe and 4',6-diamidino-2-phenylindole (DAPI) counterstaining showing microcolonies of rod-shaped bacteria (arrows). Miniature overview with DAPI counterstaining in lower left corner with marked (□) approximate area of interest. B: Magnified image with TexasRed (TXR) conjugated *P*. *aeruginosa* specific 16S rRNA probe and DAPI counterstaining. Miniature overview with DAPI counterstaining in lower left corner with marked (□) area of interest. Cluster of *P*. *aeruginosa* (arrow 1) and adjacent red blood cells (arrow 2). Display characteristics were adjusted to automatic Best Fit for red and blue color channels. Magnification with 63x/1.4 Oil objective.

Images from the confocal laser scanning examination with PNA-FISH of 5 selected thrombi with the highest relative abundance of *P*. *aeruginosa* DNA are shown in Fig 2. The examined thrombi measured approximately 2–3 mm on their long axis. Inflammatory cells, primarily polymorphonuclear leukocytes (PMNs) were abundant in the thrombi. Using the universal 16S rRNA probe, 2 of the 5 thrombi showed presence of bacterial microcolonies containing rod-shaped bacteria. The microcolonies measured up to 20 μm in diameter (Fig 2). Using a probe specific for *P*. *aeruginosa*, bacteria were found in small clusters of about 5 μm in diameter in 1 of the 5 thrombi (Fig 2).

## Discussion

In this study we examined thrombi aspirated from culprit coronary lesions of patients with STEMI, and arterial blood control samples, with use of next-generation sequencing to determine the bacterial taxonomy, and PNA-FISH to investigate the presence of intact bacteria and bacterial biofilm. We found that both thrombi and arterial blood samples contained DNA from several bacterial species. Alpha diversity analyses showed comparable diversities in both thrombi and blood. However, *P*. *aeruginosa* was significantly more abundant (ratio >30:1, p<0.0001) in thrombi than in blood.

Furthermore, microscopic examination of selected thrombi with universal and *P*. *aeruginosa*-specific PNA-FISH probes demonstrated microcolonies of intact bacteria. To the best of our knowledge, no previous microbiome study of coronary thrombi obtained *in vivo* has been conducted, and this is the first study to demonstrate high relative abundance of *P*. *aeruginosa* DNA and bacterial microcolonies within coronary thrombi.

The role of infection in AS and its clinical manifestations including myocardial infarction has been debated for decades. Early studies have focused on infectious agents such as *C*. *pneumoniae* and *H*. *pylori*, but it remains to be determined if such agents are partners in crime, or merely innocent bystanders in the atherothrombotic process. Several large randomized controlled trials have been conducted using antibiotics (primarily targeting *C*. *pneumoniae*) for secondary prevention in patients with established atherosclerotic disease, but outcomes have been mostly negative [27]. Also, a number of contemporary studies have focused on microorganisms involved in periodontal disease, and while no direct causal link with any specific oral pathogen has been determined, evidence suggests that periodontal disease is an independent risk factor for atherothrombotic disease [4, 10, 11, 19]. Indeed, presence of DNA from periodontal bacteria in aspirated coronary thrombi from patients with STEMI determined by PCR has previously been reported, but these studies used a limited range of oligonucleotide primers for typical oral pathogens only (i.e., not for *P*. *aeruginosa*) and to our knowledge, next generation sequencing and PNA-FISH data from aspirated coronary thrombi from patients with STEMI are not available [18, 19].

To examine an association between specific pathogens and coronary artery thrombosis, we analyzed the DNA sequencing results from aspirated thrombi using both a general and an oral microbiome-specific 16S database (Greengenes and HOMD, respectively). Findings on genus level were very similar with use of both databases and *Achromobacter*, *Pseudomonas*, *Sphingomonas*, and *Stenotrophomonas* were the predominant genera. The HOMD annotation yielded the highest level of species identification, and all species detected in this study are thus microorganisms that have been isolated from the human oral cavity [26]. Interestingly, none of the typical bacteria believed to be involved in more aggressive periodontal disease, e.g., *A*. *actinomycetemcomitans* and red complex bacteria (*P*. *gingivalis*, *Treponema denticola*, *Tannerella forsythia*) were identified in any of the samples. However, no specific pathogens are universally associated with periodontitis, and microbial composition of biofilms related to periodontal disease may differ considerably in composition from individual to individual and may also change over time [28]. Indeed, a previous study demonstrated that the oral microbiome of patients with chronic periodontitis only sporadically contained DNA from traditional periodontal pathogens, i.e., *P*. *gingivalis* and *A*. *actinomycetemcomitans* [28]. In that study, *A*. *xylosoxidans* was also found in the oral microbiome of patients with periodontitis [28]. Interestingly, in our study DNA from *A*. *xylosoxidans* was highly abundant in both coronary thrombi and arterial blood samples from 10/18 patients, but in the majority of the remaining patients, *A*. *xylosoxidans* constituted <0.001% of the species-specific DNA in both thrombi and blood. This strong association between the presence or absence of *A*. *xylosoxidans* in thrombi and blood, respectively, suggests that the bacteria had translocated from elsewhere (oral or other microbiomes and/or the environment) via circulating blood to atherothrombotic plaques. However, no significant correlation was observed between the presence of any detected bacterial species and self-reported periodontal disease, regular gum bleeding, or edentulism, respectively.

*P*. *aeruginosa* can be observed as part of the commensal microbiome of the oral cavity, gut, and skin in healthy humans [26, 29–32]. Though *P*. *aeruginosa* is not considered a typical pathogen in periodontal disease, *P*. *aeruginosa* has been demonstrated to be associated with the oral microbiome of patients with chronic periodontitis [29].

*P*. *aeruginosa* is a widespread rod-shaped, bacillus common to soil, moist environments, and hospitals where it is a typical opportunistic pathogen in nosocomial infections [16, 33]. *P*. *aeruginosa* and other *Pseudomonas* species have previously been isolated from human atherosclerotic tissue, and chronic infection with *P*. *aeruginosa* has been shown to exacerbate AS in a hypercholesterolemic rat model [13, 14, 34–36]. *P*. *aeruginosa* is highly adaptive and versatile and is prototypical of a multidrug-resistant organism [37]. A key component in the resistance of *P*. *aeruginosa* to both antibiotics and the immune system of the infected host is its ability to form biofilm [16]. In the form of biofilms, *P*. *aeruginosa* evades the phagocytic activity of PMNs by launching a lethal shield of the toxin di-rhamnolipid that rapidly lyses incoming PMNs [38, 39]. This releases inflammatory mediators, metallo-proteases, oxygen radicals, and extracellular DNA, that all contribute to increased inflammation and antibiotic resistance [40, 41]. Interestingly, a recent study of human carotid artery plaques revealed *Pseudomonas* species as well as bacterial biofilms within the atherosclerotic vessel wall [13]. In this study, it was also demonstrated that *P*. *aeruginosa* biofilms undergo dispersion when challenged with physiologically relevant concentrations of norepinephrine [13]. Since biofilm dispersion involves release of degradative enzymes and transition to higher bacterial growth rates this may destabilize arterial plaques and provide a mechanistic link between the hormonal stress state and risk of atherothrombotic events [13]. The presence of biofilms in atherosclerotic plaques may also explain, in part, why trials of antibiotics have failed in patients with AS as biofilms are highly resistant to systemic antibiotic therapy [16, 17, 27]. In addition to matrix-degrading proteases and di-rhamnolipid toxin, *P*. *aeruginosa* displays an impressive arsenal of other virulence factors including the potent exotoxin A, which, similarly to diphtheria toxin, inhibits protein synthesis of host cells and may remain cytotoxic long after the bacteria have been killed [16, 32]. Also, pyocyanin, a *P*. *aeruginosa* virulence factor important for quorum sensing and biofilm colonization, can elicit apoptosis of erythrocytes with phosphatidylserine externalization creating a procoagulant erythrocyte phenotype and contributing to a prothrombotic microenvironment [32, 42]. Taken together, our current analyses of aspirated coronary thrombi from patients with STEMI therefore considerably expand the accumulating evidence that *P*. *aeruginosa* and biofilms can play a pathogenic role in development of atherothrombotic plaques and their clinical manifestations.

Some limitations apply to the results of this study, especially the small sample size, the lack of oral examination and individual mapping of oral microbiomes of patients, and the inherent limitations of the applied analytical techniques. Importantly, our DNA sequencing data were supported by the PNA-FISH results. Although we did not confirm bacterial viability with culture experiments, the recording of distinct fluorescent signals in biofilm-like aggregates are highly suggestive of viable bacteria, since only live cells contain intact rRNA that enables probe-hybridization. Moreover, our PNA-FISH examination was limited by the availability of thrombus material, i.e. only approximately half of the each thrombus was cast in paraffin (the rest was DNA-amplified and sequenced) and only three serial 2 μm thick sections of each thrombus were examined with universal and *P*. *aeruginosa* specific 16S probes, respectively. Therefore, we believe that the extent of *P*. *aeruginosa* microcolonies in thrombi may have been underestimated. Of note, although the diameter of detected aggregates did not exceed 20 μm in our study, biofilm have generally been defined as bacterial aggregates as small as 5 μm in diameter [17].

In conclusion, we observed that DNA from *P*. *aeruginosa* was significantly more abundant in aspirated coronary thrombi than in arterial blood from patients with STEMI. Microcolonies of bacteria were detected within thrombi in biofilm-like aggregates. The role of *P*. *aeruginosa* infection and biofilms in atherothrombotic coronary culprit lesions of patients with STEMI warrants further study.

## Supporting Information

S1 Dataset Numbers of DNA reads per species for all samples using the Human Oral Microbiome Database (HOMD) annotation. (XLSX)

S1 File **Fig A. Venn diagram showing the distribution of operational taxonomic units (OTUs) in aspirated coronary thrombi and arterial blood samples of patients with ST-segment elevation myocardial infarction**. **Fig B. Alpha diversity of bacteria in aspirated coronary thrombi and arterial blood of patients with ST-segment elevation myocardial infarction**. A: Chao index. B: Shannon index. C: Simpsons index. Alpha diversity is displayed as means and ranges. No statistical differences were observed. **Fig C. Predominant bacterial genera in aspirated coronary thrombi and arterial blood of patients with ST-segment elevation myocardial infarction**. Data were based on Greengenes annotation. **Table A. Bacterial genus level identification in aspirated coronary thrombi and arterial blood from patients with ST-segment elevation myocardial infaction using the Greengenes database**. Complete list of bacterial genera expressed as their relative (mean±SD) abundance in thrombi and blood samples. **Table B. Bacterial genus level identification in aspirated coronary thrombi and arterial blood from patients with ST-segment elevation myocardial infaction using the Human Oral Microbiome Database (HOMD)**. Complete list of bacterial genera expressed as their relative (mean±SD) abundance in thrombi and blood samples. **Table C. Bacterial species level identification in aspirated coronary thrombi and arterial blood from patients with ST-segment elevation myocardial infaction using the Human Oral Microbiome Database (HOMD)**. Complete list of bacterial species expressed as their relative (mean±SD) abundance in thrombi and blood samples.
(DOCX)
