# Supplementary material for: Pseudomonas aeruginosa Microcolonies in Coronary Thrombi from Patients with ST-Segment Elevation Myocardial Infarction
Source: PLoS One. 2016 Dec 28;11(12):e0168771. doi: 10.1371/journal.pone.0168771 (PMC5193428; doi:10.1371/journal.pone.0168771)
Supplement: S1 File — Fig A. Venn diagram showing the distribution of operational taxonomic units (OTUs) in aspirated coronary thrombi and arterial blood samples of patients with ST-segment elevation myocardial infarction. Fig B. Alpha diversity of bacteria in aspirated coronary thrombi and arterial blood of patients with ST-segment elevation myocardial infarction. A: Chao index. B: Shannon index. C: Simpsons index. Alpha diversity is displayed as means and ranges. No statistical differences were observed. Fig C. Predominant bacterial genera in aspirated coronary thrombi and arterial blood of patients with ST-segment elevation myocardial infarction. Data were based on Greengenes annotation. Table A. Bacterial genus level identification in aspirated coronary thrombi and arterial blood from patients with ST-segment elevation myocardial infaction using the Greengenes database. Complete list of bacterial genera expressed as their relative (mean±SD) abundance in thrombi and blood samples. Table B. Bacterial genus level identification in aspirated coronary thrombi and arterial blood from patients with ST-segment elevation myocardial infaction using the Human Oral Microbiome Database (HOMD). Complete list of bacterial genera expressed as their relative (mean±SD) abundance in thrombi and blood samples. Table C. Bacterial species level identification in aspirated coronary thrombi and arterial blood from patients with ST-segment elevation myocardial infaction using the Human Oral Microbiome Database (HOMD). Complete list of bacterial species expressed as their relative (mean±SD) abundance in thrombi and blood samples. (DOCX) [file pone.0168771.s002.docx]

**Supporting Information**

**Table A**

|  | Thrombi | | Blood | |  |
| --- | --- | --- | --- | --- | --- |
| Taxon | Mean | SD | Mean | SD | Difference |
| *Achromobacter* | 24.72% | 23.35% | 31.16% | 23.79% | -6.44% |
| *Pseudomonas* | 32.06% | 27.24% | 7.21% | 5.04% | 24.85% |
| *Sphingomonas* | 6.69% | 4.92% | 9.98% | 5.44% | -3.30% |
| *Stenotrophomonas* | 5.90% | 5.96% | 7.12% | 5.87% | -1.22% |
| *Janthinobacterium* | 4.02% | 6.70% | 4.00% | 6.64% | 0.02% |
| *Halomonas* | 2.00% | 3.42% | 3.90% | 7.54% | -1.90% |
| *Burkholderia* | 2.72% | 1.69% | 3.16% | 1.78% | -0.44% |
| *Phyllobacterium* | 2.52% | 4.52% | 2.99% | 5.61% | -0.47% |
| *Paracoccus* | 2.12% | 7.06% | 2.76% | 7.04% | -0.63% |
| *Corynebacterium* | 0.48% | 1.50% | 3.45% | 7.36% | -2.97% |
| *Ralstonia* | 1.16% | 1.46% | 1.48% | 1.54% | -0.32% |
| *Micrococcus* | 0.49% | 1.25% | 2.16% | 6.11% | -1.67% |
| *Bacillus* | 0.94% | 1.56% | 1.01% | 1.68% | -0.08% |
| *Phormidium* | 1.48% | 6.28% | 0.00% | 0.00% | 1.48% |
| *Bradyrhizobium* | 0.58% | 0.70% | 0.58% | 0.72% | -0.01% |
| *Meiothermus* | 0.60% | 0.73% | 0.47% | 0.52% | 0.14% |
| *Methylobacterium* | 0.38% | 0.67% | 0.48% | 1.42% | -0.10% |
| *Porphyromonas* | 0.00% | 0.00% | 0.89% | 3.77% | -0.89% |
| *Streptococcus* | 0.09% | 0.20% | 0.61% | 2.34% | -0.52% |
| *Anaerococcus* | 0.02% | 0.08% | 0.67% | 2.12% | -0.65% |
| *Staphylococcus* | 0.12% | 0.28% | 0.49% | 0.87% | -0.37% |
| *Enhydrobacter* | 0.12% | 0.43% | 0.39% | 1.45% | -0.27% |
| *Acinetobacter* | 0.41% | 0.92% | 0.04% | 0.12% | 0.37% |
| *Nesterenkonia* | 0.17% | 0.35% | 0.22% | 0.46% | -0.05% |
| *Peptoniphilus* | 0.23% | 0.88% | 0.16% | 0.68% | 0.07% |
| *Sediminibacterium* | 0.22% | 0.50% | 0.10% | 0.27% | 0.12% |
| *Finegoldia* | 0.00% | 0.00% | 0.33% | 1.31% | -0.33% |
| *Rhizobium* | 0.07% | 0.23% | 0.25% | 0.60% | -0.17% |
| *Virgibacillus* | 0.15% | 0.21% | 0.15% | 0.37% | 0.00% |
| Other | 0.68% | 0.72% | 2.13% | 2.92% | -1.45% |
| Unclassified | 8.84% | 9.50% | 11.64% | 10.66% | -2.79% |
| **Table A. Bacterial genus level identification in aspirated coronary thrombi and arterial blood from patients with ST-segment elevation myocardial infaction using the Greengenes database.** Complete list of bacterial genera expressed as their relative (mean±SD) abundance in thrombi and blood samples. | | | | | |

**Table B**

|  | Thrombi | | Blood | |  |
| --- | --- | --- | --- | --- | --- |
| Taxon | Mean | SD | Mean | SD | Difference |
| *Achromobacter* | 24.72% | 23.34% | 31.16% | 23.79% | -6.4437% |
| *Unmatched* | 18.19% | 21.46% | 26.17% | 27.06% | -7.9777% |
| *Pseudomonas* | 32.23% | 27.13% | 7.26% | 5.08% | 24.9741% |
| *Sphingomonas* | 6.93% | 4.84% | 10.18% | 5.44% | -3.2486% |
| *Stenotrophomonas* | 5.90% | 5.96% | 7.12% | 5.87% | -1.2237% |
| *Burkholderia* | 2.72% | 1.69% | 3.16% | 1.78% | -0.4397% |
| *Rhodobacter* | 1.96% | 7.06% | 2.09% | 6.73% | -0.1341% |
| *Corynebacterium* | 0.46% | 1.41% | 3.26% | 7.22% | -2.8030% |
| *Ralstonia* | 1.16% | 1.46% | 1.51% | 1.51% | -0.3454% |
| *Brevundimonas* | 1.24% | 1.85% | 1.24% | 2.13% | -0.0031% |
| *Bacillus* | 0.94% | 1.56% | 1.02% | 1.68% | -0.0750% |
| *Klebsiella* | 0.90% | 1.95% | 0.97% | 1.71% | -0.0690% |
| *Bradyrhizobium* | 0.58% | 0.70% | 0.60% | 0.78% | -0.0245% |
| *Afipia* | 0.48% | 0.66% | 0.54% | 1.40% | -0.0582% |
| *Streptococcus* | 0.09% | 0.20% | 0.61% | 2.34% | -0.5227% |
| *Anaerococcus* | 0.02% | 0.08% | 0.67% | 2.12% | -0.6453% |
| *Staphylococcus* | 0.12% | 0.28% | 0.49% | 0.87% | -0.3702% |
| *Moraxella* | 0.12% | 0.43% | 0.39% | 1.45% | -0.2735% |
| *Kocuria* | 0.17% | 0.34% | 0.28% | 0.49% | -0.1057% |
| *Acinetobacter* | 0.41% | 0.92% | 0.04% | 0.12% | 0.3736% |
| *Peptoniphilus* | 0.23% | 0.88% | 0.17% | 0.70% | 0.0633% |
| *Finegoldia* | 0.00% | 0.00% | 0.34% | 1.31% | -0.3361% |
| *Flavobacteriales_[G-2]* | 0.23% | 0.50% | 0.10% | 0.27% | 0.1219% |
| *Bergeyella* | 0.02% | 0.06% | 0.13% | 0.38% | -0.1096% |
| *Caulobacter* | 0.03% | 0.10% | 0.05% | 0.19% | -0.0160% |
| *Clostridiales_[F-2][G-1]* | 0.01% | 0.06% | 0.06% | 0.24% | -0.0431% |
| *Neisseria* | 0.00% | 0.00% | 0.06% | 0.18% | -0.0551% |
| *Actinomyces* | 0.00% | 0.00% | 0.05% | 0.17% | -0.0450% |
| *Dietzia* | 0.01% | 0.02% | 0.04% | 0.15% | -0.0331% |
| *Desulfovibrio* | 0.00% | 0.00% | 0.04% | 0.16% | -0.0372% |
| *Kytococcus* | 0.00% | 0.01% | 0.03% | 0.11% | -0.0312% |
| *Microbacterium* | 0.00% | 0.00% | 0.03% | 0.13% | -0.0325% |
| *Alloiococcus* | 0.00% | 0.00% | 0.03% | 0.12% | -0.0294% |
| *Dialister* | 0.00% | 0.00% | 0.03% | 0.11% | -0.0267% |
| *Rhodocyclus* | 0.00% | 0.00% | 0.02% | 0.05% | -0.0218% |
| *Mycoplasma* | 0.00% | 0.02% | 0.01% | 0.05% | -0.0077% |
| *Prevotella* | 0.00% | 0.00% | 0.01% | 0.03% | -0.0081% |
| *Other* | 0.01% | 0.03% | 0.00% | 0.00% | 0.0078% |
| *Terrahaemophilus* | 0.01% | 0.03% | 0.00% | 0.00% | 0.0060% |
| *Veillonella* | 0.00% | 0.01% | 0.00% | 0.02% | -0.0025% |
| *Lactobacillus* | 0.01% | 0.02% | 0.00% | 0.00% | 0.0053% |
| *Gemella* | 0.00% | 0.00% | 0.00% | 0.01% | -0.0023% |
| *Bifidobacterium* | 0.00% | 0.01% | 0.00% | 0.00% | 0.0025% |
| *Leptothrix* | 0.00% | 0.00% | 0.00% | 0.01% | -0.0020% |
| *Propionibacterium* | 0.00% | 0.00% | 0.00% | 0.00% | 0.0009% |
| *Clostriales_[F-3][G-1]* | 0.00% | 0.00% | 0.00% | 0.01% | -0.0017% |
| *Flavobacteriales_[G-1]* | 0.00% | 0.00% | 0.00% | 0.00% | -0.0008% |
| *Granulicatella* | 0.00% | 0.00% | 0.00% | 0.00% | 0.0014% |
| *Bacteroides* | 0.00% | 0.00% | 0.00% | 0.00% | -0.0006% |
| *Porphyromonas* | 0.00% | 0.00% | 0.00% | 0.00% | -0.0003% |
| **Table B. Bacterial genus level identification in aspirated coronary thrombi and arterial blood from patients with ST-segment elevation myocardial infaction using the Human Oral Microbiome Database (HOMD)**. Complete list of bacterial genera expressed as their relative (mean±SD) abundance in thrombi and blood samples. | | | | | |

**Table C**

|  | Thrombi | | Blood | |  |
| --- | --- | --- | --- | --- | --- |
| Taxon | Mean % | SD % | Mean% | SD % | Diff. % |
| Unclassified | 23.71 | 23.45 | 33.42 | 28.13 | -9.71 |
| Achromobacter_xylosoxidans_oral_taxon_343 | 24.72 | 23.35 | 28.04 | 23.79 | -3.32 |
| Pseudomonas_aeruginosa_oral_taxon_536 | 28.23 | 29.26 | 0.89 | 2.77 | 27.34 |
| Sphingomonas_sp._oral_taxon_003 | 6.69 | 4.92 | 8.98 | 5.44 | -2.30 |
| Stenotrophomonas_maltophilia_oral_taxon_663 | 5.90 | 5.96 | 6.41 | 5.87 | -0.51 |
| Burkholderia_cepacia_oral_taxon_571 | 2.72 | 1.69 | 2.85 | 1.78 | -0.12 |
| Rhodobacter_capsulatus_oral_taxon_857 | 1.96 | 7.06 | 1.88 | 6.73 | 0.08 |
| Ralstonia_pickettii_oral_taxon_854 | 1.16 | 1.46 | 1.33 | 1.54 | -0.17 |
| Brevundimonas_diminuta_oral_taxon_590 | 1.24 | 1.85 | 1.12 | 2.13 | 0.12 |
| Klebsiella_pneumoniae_oral_taxon_731 | 0.90 | 1.95 | 0.87 | 1.71 | 0.03 |
| Bradyrhizobium_elkanii_oral_taxon_597 | 0.58 | 0.70 | 0.54 | 0.78 | 0.04 |
| Afipia_sp._genomospecies_8_oral_taxon_636 | 0.48 | 0.66 | 0.48 | 1.41 | 0.00 |
| Staphylococcus_caprae_oral_taxon_567 | 0.12 | 0.28 | 0.44 | 0.87 | -0.32 |
| ActiCorynebacterium_diphtheriae_oral_taxon_591 | 0.07 | 0.17 | 0.46 | 1.50 | -0.39 |
| Corynebacterium_mucifaciens_oral_taxon_835 | 0.08 | 0.27 | 0.43 | 1.26 | -0.35 |
| Moraxella_osloensis_oral_taxon_711 | 0.12 | 0.43 | 0.35 | 1.45 | -0.23 |
| Kocuria_sp._oral_taxon_189 | 0.17 | 0.34 | 0.25 | 0.49 | -0.08 |
| Acinetobacter_baumannii_oral_taxon_554 | 0.41 | 0.92 | 0.04 | 0.12 | 0.38 |
| Finegoldia_magna_oral_taxon_662 | 0.00 | 0.00 | 0.30 | 1.31 | -0.30 |
| Peptoniphilus_indolicus_oral_taxon_840 | 0.23 | 0.88 | 0.09 | 0.43 | 0.14 |
| Flavobacteriales_[G-2]_sp._oral_taxon_320 | 0.23 | 0.50 | 0.09 | 0.27 | 0.13 |
| Bergeyella_sp._oral_taxon_422 | 0.02 | 0.06 | 0.12 | 0.38 | -0.10 |
| Sphingomonas_sp._oral_taxon_007 | 0.09 | 0.16 | 0.03 | 0.10 | 0.06 |
| Anaerococcus_tetradius_oral_taxon_788 | 0.00 | 0.00 | 0.11 | 0.53 | -0.11 |
| Sphingomonas_sp._oral_taxon_006 | 0.00 | 0.01 | 0.11 | 0.29 | -0.11 |
| Caulobacter_sp._oral_taxon_002 | 0.03 | 0.10 | 0.04 | 0.19 | -0.01 |
| Clostridiales_[F-2][G-1]_sp._oral_taxon_075 | 0.01 | 0.06 | 0.05 | 0.24 | -0.04 |
| Dietzia_sp._oral_taxon_368 | 0.00 | 0.02 | 0.03 | 0.15 | -0.03 |
| Desulfovibrio_fairfieldensis_oral_taxon_605 | 0.00 | 0.00 | 0.03 | 0.16 | -0.03 |
| Kytococcus_sedentarius_oral_taxon_855 | 0.00 | 0.01 | 0.03 | 0.11 | -0.03 |
| Streptococcus_mutans_oral_taxon_686 | 0.03 | 0.11 | 0.01 | 0.04 | 0.02 |
| Pseudomonas_pseudoalcaligenes_oral_taxon_740 | 0.03 | 0.10 | 0.00 | 0.01 | 0.03 |
| Pseudomonas_stutzeri_oral_taxon_477 | 0.03 | 0.12 | 0.00 | 0.00 | 0.03 |
| Microbacterium_sp._oral_taxon_186 | 0.00 | 0.00 | 0.03 | 0.12 | -0.03 |
| Alloiococcus_otitis_oral_taxon_831 | 0.00 | 0.00 | 0.03 | 0.13 | -0.03 |
| Streptococcus_salivarius_oral_taxon_755 | 0.01 | 0.03 | 0.02 | 0.06 | -0.01 |
| Ralstonia_sp._oral_taxon_027 | 0.00 | 0.00 | 0.03 | 0.12 | -0.03 |
| Rhodocyclus_sp._oral_taxon_028 | 0.00 | 0.00 | 0.02 | 0.05 | -0.02 |
| Mycoplasma_fermentans_oral_taxon_607 | 0.00 | 0.02 | 0.01 | 0.05 | -0.01 |
| Afipia_sp._genomospecies_4_oral_taxon_652 | 0.00 | 0.00 | 0.01 | 0.03 | -0.01 |
| Prevotella_buccalis_oral_taxon_562 | 0.00 | 0.00 | 0.01 | 0.03 | -0.01 |
| Terrahaemophilus_aromaticivorans_oral_taxon_826 | 0.01 | 0.03 | 0.00 | 0.00 | 0.01 |
| Bacillus_subtilis_oral_taxon_468 | 0.00 | 0.02 | 0.00 | 0.00 | 0.00 |
| Gemella_morbillorum_oral_taxon_046 | 0.00 | 0.00 | 0.00 | 0.01 | 0.00 |
| Bacillus_clausii_oral_taxon_045 | 0.00 | 0.00 | 0.00 | 0.01 | 0.00 |
| Propionibacterium_acnes_oral_taxon_530 | 0.00 | 0.00 | 0.00 | 0.00 | 0.00 |
| Clostriales_[F-3][G-1]_sp._oral_taxon_876 | 0.00 | 0.00 | 0.00 | 0.01 | 0.00 |
| Granulicatella_adiacens_oral_taxon_534 | 0.00 | 0.00 | 0.00 | 0.00 | 0.00 |
| Porphyromonas_asaccharolytica_oral_taxon_547 | 0.00 | 0.00 | 0.00 | 0.00 | 0.00 |
| Bacteroides_ureolyticus_oral_taxon_842 | 0.00 | 0.00 | 0.00 | 0.00 | 0.00 |
| Mobiluncus_mulieris_oral_taxon_830 | 0.00 | 0.00 | 0.00 | 0.00 | 0.00 |
| Mycobacterium_neoaurum_oral_taxon_692 | 0.00 | 0.00 | 0.00 | 0.00 | 0.00 |
| Mycobacterium_tuberculosis_oral_taxon_822 | 0.00 | 0.00 | 0.00 | 0.00 | 0.00 |
| Peptoniphilus_sp._oral_taxon_375 | 0.00 | 0.00 | 0.00 | 0.00 | 0.00 |
| Prevotella_sp._oral_taxon_313 | 0.00 | 0.00 | 0.00 | 0.00 | 0.00 |
| Xanthomonas_sp._oral_taxon_037 | 0.00 | 0.00 | 0.00 | 0.00 | 0.00 |
| **Table C. Bacterial species level identification in aspirated coronary thrombi and arterial blood from patients with ST-segment elevation myocardial infaction using the Human Oral Microbiome Database (HOMD).** Complete list of bacterial species expressed as their relative (mean±SD) abundance in thrombi and blood samples. | | | | | |

**Fig A**

**
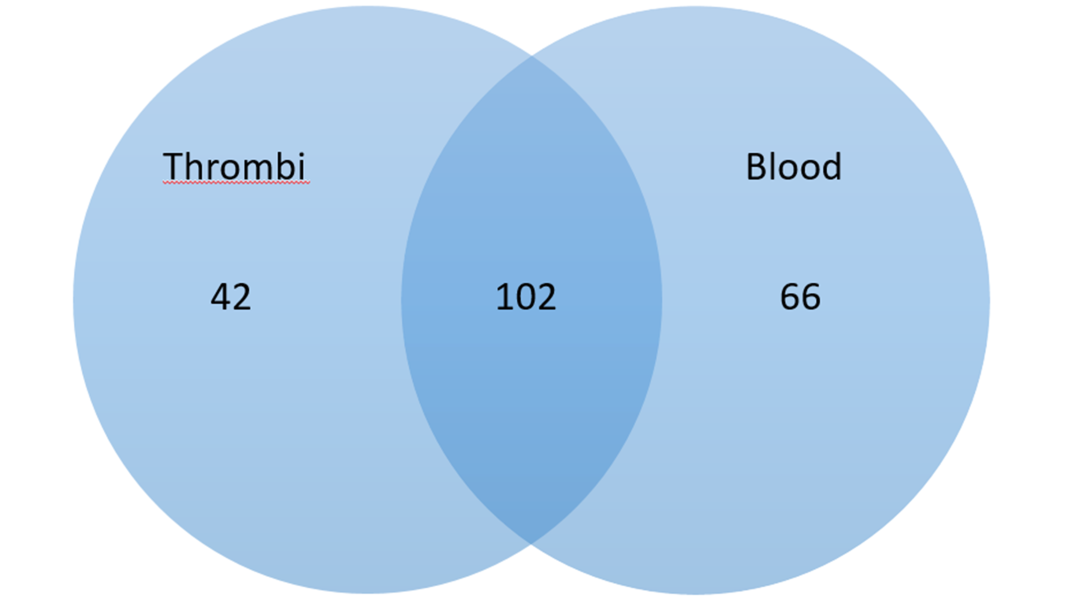
**

**Fig A. Venn diagram showing the distribution of operational taxonomic units (OTUs) in aspirated coronary thrombi and arterial blood samples of patients with ST-segment elevation myocardial infarction**.

**Fig B**

**
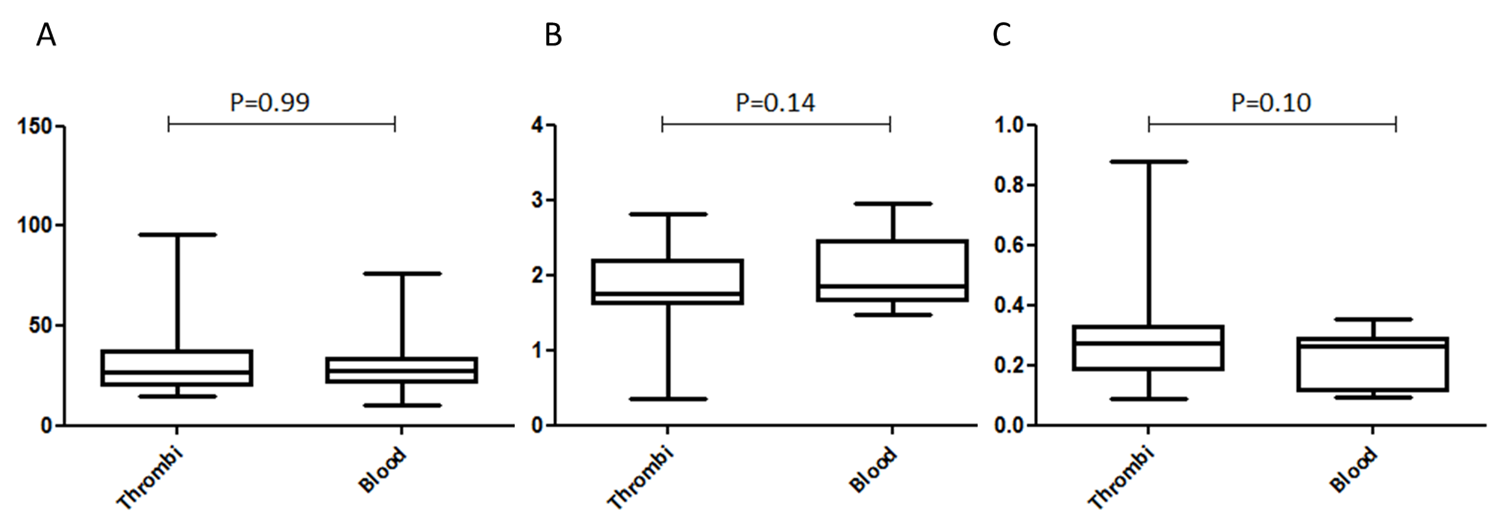
**

**Fig B. Alpha diversity of bacteria in aspirated coronary thrombi and arterial blood of patients with ST-segment elevation myocardial infarction**. A: Chao index. B: Shannon index. C: Simpsons index. Alpha diversity is displayed as means and ranges. No statistical differences were observed.

**Fig C**


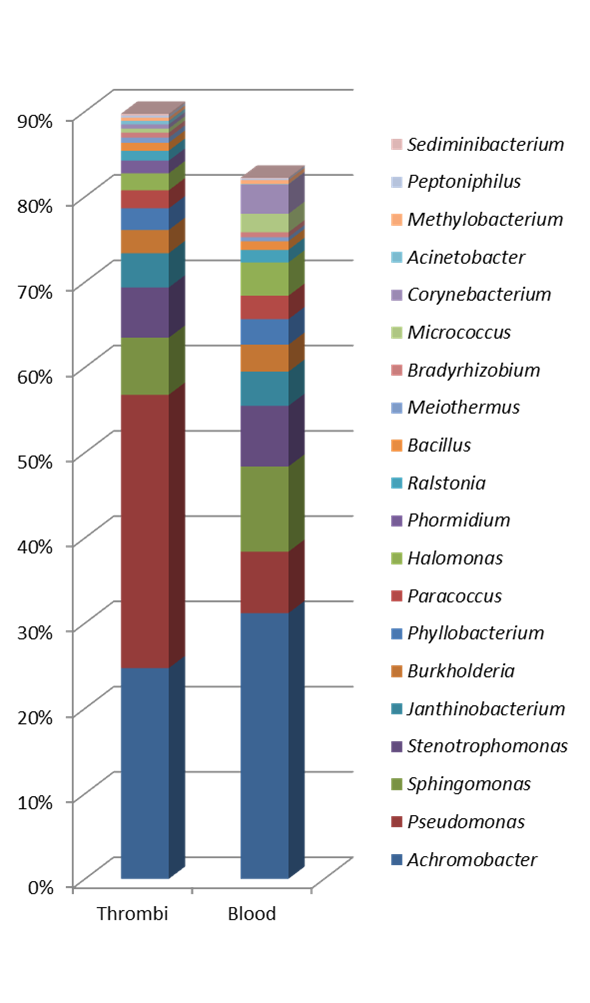


**Fig C. Predominant bacterial genera in aspirated coronary thrombi and arterial blood of patients with ST-segment elevation myocardial infarction**. Data were based on Greengenes annotation.
